# There is (still) too much aluminium in infant formulas

**DOI:** 10.1186/1471-2431-10-63

**Published:** 2010-08-31

**Authors:** Shelle-Ann M Burrell, Christopher Exley

**Affiliations:** 1 Life Sciences, Huxley Building, Keele University, Staffordshire, UK; 2 The Birchall Centre, Lennard-Jones Laboratories, Keele University, Staffordshire, UK

## Abstract

**Background:**

Infant formulas are sophisticated milk-based feeds for infants which are used as a substitute for breast milk. Historically they are known to be contaminated by aluminium and in the past this has raised health concerns for exposed infants. We have measured the aluminium content of a number of widely used infant formulas to determine if their contamination by aluminium and consequent issues of child health persists.

**Methods:**

Samples of ready-made milks and powders used to make milks were prepared by microwave digestion of acid/peroxide mixtures and their aluminium content determined by THGA.

**Results:**

The concentration of aluminium in ready-made milks varied from *ca *176 to 700 μg/L. The latter concentration was for a milk for preterm infants. The aluminium content of powders used to make milks varied from *ca *2.4 to 4.3 μg/g. The latter content was for a soya-based formula and equated to a ready-to-drink milk concentration of 629 μg/L. Using the manufacturer's own guidelines of formula consumption the average daily ingestion of aluminium from infant formulas for a child of 6 months varied from *ca *200 to 600 μg of aluminium. Generally ingestion was higher from powdered as compared to ready-made formulas.

**Conclusions:**

The aluminium content of a range of well known brands of infant formulas remains high and particularly so for a product designed for preterm infants and a soya-based product designed for infants with cow's milk intolerances and allergies. Recent research demonstrating the vulnerability of infants to early exposure to aluminium serves to highlight an urgent need to reduce the aluminium content of infant formulas to as low a level as is practically possible.

## Background

Infant formulas are milk-based feeds for infants which have been developed as alternatives to breast milk. Though cow's milk is the main ingredient of many infant formulas they are sophisticated products which have been designed to meet the specific nutritional needs of children from babies born pre-term through to infants of several years of age [1]. There are also non-cow's milk-based formulas, often made from soya, for infants with intolerances or allergies to cow's milk [2].

There has been a long and significant history documenting the contamination of infant formulas by aluminium [3-9] and consequent health effects in children [10-13]. Through these and other publications manufacturers of infant formulas have been made fully aware of the potentially compounded issue of both the contamination by aluminium and the heightened vulnerability, from the point of view of a newborn's developing physiology, of infants fed such formulas. There have been similar warnings over several decades in relation to aluminium toxicity and parenteral nutrition of preterm and term infants [14-17]. To these ends the expectation would be that the aluminium content of current infant formulas would at the very least be historically low and at best would be as low as might be achieved for a processed product. We have tested this premise and we have found that the aluminium content of a range of branded infant formulas remains too high.

## Methods

We have chosen 15 different branded infant formula products. These include powdered and ready-made liquid formulas based on cow's milk and a soya-based product. The categories of formulas included those for preterm babies, stage one (0-6 months) and stage two (6 months plus) infants. All products were stored according to the manufacturer's instructions. Products were sampled directly from their packaging to avoid extraneous contamination. Ready-made liquid products were shaken between each sampling.

Homogenates of each product were prepared by microwave digestion (Mars Xpress, CEM) using a 50/50 mixture of 14 M HNO_3 _and 30% *w/v *H_2_O_2_. Homogenates were diluted as required in ultra pure water (conductivity < 0.067 μS/cm) to give clear samples. The aluminium content of samples was measured by Transversley Heated Graphite Atomiser (THGA; Analyst 600, PE Life Sciences) using an analytical programme developed in our laboratory. Five replicate samples were prepared for each of the products. Each sample was measured 3 times and its mean was accepted if the %RSD was <10%.

## Results

### The aluminium content of ready-made milk formulas

The mean aluminium content of ready-made milk formulas ranged from *ca *176 μg/L (Hipp Organic Growing-Up Milk) to *ca *700 μg/L (Cow & Gate Nutriprem 1) (Table 1). Two products (Cow & Gate Growing-Up Milk and Cow & Gate Nutriprem 1) presented a wide range of values which suggested an inhomogeneous distribution of aluminium in these products. Generally Cow & Gate products had higher contents of aluminium than the other brands tested.

**Table 1:** The aluminium content of ready-made (RM) milk infant formulas.

Commercial Name of Product N = 5	[Al] μg/L Mean (SD)	[Al] μg/L Range
**Sma First Infant Milk**	267.9 (40.9)	210.1-322.5

**Sma Follow-On Milk**	245.8 (59.0)	174.5-309.8

**Cow & Gate First Infant Milk**	338.8 (34.8)	293.0-371.0

**Hipp Organic Growing-Up Milk**	175.5 (34.7)	131.4-236.8

**Aptamil Follow-On Milk**	296.1 (13.9)	279.3-314.2

**Cow & Gate Follow-On Milk**	303.7 (10.8)	285.3-316.8

**Cow & Gate Growing-Up Milk**	430.0 (214.8)	285.3-856.5

**Cow & Gate Nutriprem 1**	700.4 (93.6)	602.5-863.0

### The aluminium content of powders used to make milk formulas

The mean aluminium content of milk powders ranged from *ca *2.4 μg Al/g powder (Sma First Infant Milk) to *ca *4.3 μg/g (Sma Wysoy Soya Infant Formula) (Table 2). The range of values for the 5 replicates of each sample was high for almost all products (for example, 1.7 - 10.8 μg/g for Cow & gate Follow-On Milk) which suggested that aluminium was not evenly distributed within the milk powders. When the aluminium content of the powders were used to make reliable estimates of their aluminium content as ready-to-drink milks the values ranged from *ca *333 to 629 μg/L (Table 2). In general, the aluminium content of formulas prepared from powdered milks were significantly higher than ready-made milks (for example, 296.1 and 592.4 μg/L for Aptamil Follow-On Milk 'ready-made' and 'powdered' milks respectively).

**Table 2:** The aluminium content of milk powders (P) used in formulas.

Commercial Name of Product N = 5	[Al] μg/g Mean (SD)	[Al] μg/g Range	[Al] μg/L*
**Sma Wysoy Soya Infant Formula**	4.3 (1.0)	3.7-6.0	629.0

**Sma First Infant Milk**	2.4 (1.4)	1.3-4.6	333.3

**Hipp Organic Follow-On Milk**	3.6 (1.6)	2.1-6.3	500.0

**Hipp Organic Good Night Milk**	2.9 (1.5)	1.7-5.5	406.0

**Cow & Gate First Infant Milk**	2.8 (0.6)	1.8-3.5	424.0

**Hipp Organic First Infant Milk**	2.7 (1.3)	0.2-4.2	394.4

**Aptamil Follow-On Milk**	3.1 (0.5)	2.3-3.8	592.4

**Cow & Gate Follow-On Milk**	2.5 (3.4)	1.7-10.8	477.8

*Based upon manufacturer's instructions for preparing the milk.

### The average daily ingestion of aluminium in infant formulas

The average ingestion of aluminium in infant formulas for children aged 6 months ranged from 206 (Sma Follow-On Milk RM) to 592 (eg. Sma Wysoy Soya Infant Formula P) μg Al per 24 h period (Table 3). All values were determined based upon manufacturer's guides to age-related consumption. Ingestion is predicted to be higher from formulas prepared from powders than ready-made milk formulas (for example, 296 and 532 μg Al/24 h for Aptamil Follow-On Milk 'ready-made' and 'powdered' milks respectively). Generally the greatest exposure to aluminium was through the Hipp Organic products and the Sma soya-based product.

**Table 3:** The daily ingestion of aluminium by infants at 6 months of age based upon the mean aluminium content of the product and the manufacturers recommended feeding volumes.

Commercial Name of Product RM-ready made; P-powdered	Al ingested from product **μg Al/24 h period**.
**Sma First Infant Milk RM**	224

**Sma First Infant Milk P**	323

**Sma Follow-On Milk RM**	206

**Sma Wysoy Soya Infant Formula P**	592

**Cow & Gate First Infant Milk RM**	285

**Cow & Gate First Infant Milk P**	385

**Cow & Gate Follow-On Milk RM**	301

**Cow & Gate Follow-On Milk P**	429

**Cow & Gate Growing-Up Milk RM**	107 (at 12 months)

**Cow & Gate Nutriprem 1 RM**	112-263*

**Aptamil Follow-On Milk RM**	296

**Aptamil Follow-On Milk P**	532

**Hipp Organic Growing-Up Milk RM**	88 (at 12 months)

**Hipp Organic First Infant Milk P**	380

**Hipp Organic Follow-On Milk P**	592

**Hipp Organic Good Night Milk P**	477

*Values are for preterm infants of a very low initial body weight (< 1 kg) up to term (*ca *2.5 kg)

...

## Discussion

Commercially available branded infant formulas used by literally millions of parents to feed children of up to 12 months plus of age are still significantly contaminated with aluminium. The concentrations of aluminium in the milk formulas varied from *ca *200 - 700 μg/L and would result in the ingestion of up to 600 μg of aluminium per day. The suggestion is that these products are 'contaminated' with aluminium as each of the manufacturers insist that aluminium is not knowingly added to their products. Milk formulas prepared from powders contained significantly more aluminium than their equivalent ready-made product. Aluminium products are used extensively in food processing [18], for example, as anti-caking agents, though there is no indication that they are being used in this way in powdered milk formulas. The likelihood is that many of the individual constituents of the formulas are contaminated with aluminium [19,20]. The sources of such contamination are myriad though would probably include equipment used in both processing and storing of bulk products. In addition many of the formulas were packaged for sale using aluminium-based materials. The high content of aluminium in the soya-based formula probably reflects its prior accumulation in the soybean plant and the known aluminium tolerance of some soybean cultivars that are grown on acid soils [21]. Previous research has also highlighted higher contents of aluminium in soya-based infant formulas [9].

The aluminium content of infant formulas measured herein are not significantly different to historical values and this lack of improvement in lowering their content suggests either that the manufacturers are not monitoring the aluminium content of their products or that the manufacturers are not concerned at these levels of contamination. While it is the case that the present levels of aluminium in infant formulas have not been shown to cause adverse effects in healthy infants it is also the case that there have not been any clinical studies which refute such as a possibility. Previous research has highlighted the potential toxicity of aluminium in infants with confounding disorders (including, prematurity, poor renal function and gastrointestinal disease) and fed infant formulas [10-13] and these studies when viewed alongside aluminium's known connections with medicine and human disease [22] should at least deter complacency concerning this issue. It is widely accepted that the not fully developed physiologies of infant's gastrointestinal tract, kidneys and blood-brain barrier may predispose them to aluminium toxicity [10,11,16,23,24] and while there are no definitive links between aluminium exposure through infant formulas and immediate or delayed toxicity in healthy infants this neither should not nor does not preclude such as a possibility. The widespread use of infant formulas would necessitate that any attempt at an epidemiological study would require a Herculean effort even with well-defined levels of exposure and quantifiable end-points. However, there are clear links between toxicity in infants and parenteral exposure to aluminium. For example, parenteral exposure of preterm infants to *ca *55 μg Al/kg body weight/day, which is a level of systemic exposure to aluminium which is possible from regular feeding of infant formulas over periods of weeks, resulted, at 18 months of age, in neurodevelopmental effects [15] and, in the same cohort of children 15 years later, in significant affects upon bone health [17]. The authors concluded, with good reason, that the potential long-term consequences of early aluminium exposure deserve renewed attention [17]. The aluminium content of infant formulas is between 10 and 40 times higher than the aluminium content of breast milk, (usually ca 15 - 30 μg/L [7]), and will contribute significantly towards the body burden of aluminium in infants. It is clear that aluminium in infant formulas is a significant component of early life exposure to this ubiquitous contaminant and as such every effort should be made by manufacturers to reduce the aluminium content of these products to an achievable practical minimum while at the same time manufacturers should be compelled to indicate the level of contamination by aluminium on the packaged product.

## Conclusions

Infant formulas are integral to the nutritional requirements of preterm and term infants. While it has been known for decades that infant formulas are contaminated with significant amounts of aluminium there is little evidence that manufacturers consider this to be a health issue. Aluminium is non-essential [25] and is linked to human disease [22]. There is evidence of both immediate and delayed toxicity in infants, and especially preterm infants, exposed to aluminium and it is our contention that there is still too much aluminium in infant formulas.

## Competing interests

The authors declare that they have no competing interests.

## Authors' contributions

CE conceived, designed and supervised the study and wrote the manuscript.

SAB carried out the majority of the experiments and helped with the manuscript.

Both authors have read and approved the manuscript.

## Pre-publication history

The pre-publication history for this paper can be accessed here:

http://www.biomedcentral.com/1471-2431/10/63/prepub
